# Association of accelerometer-measured physical activity with kidney function in a Japanese population: the DOSANCO Health Study

**DOI:** 10.1186/s12882-021-02635-0

**Published:** 2022-01-03

**Authors:** Sachiko Sasaki, Koshi Nakamura, Shigekazu Ukawa, Emiko Okada, Shiho Amagasa, Shigeru Inoue, Takashi Kimura, Aya Yoshimura, Aya Tanaka, Takafumi Nakagawa, Akihiro Imae, Akiko Tamakoshi

**Affiliations:** 1 Department of Public Health, Hokkaido University Faculty of Medicine, Kita 15, Nishi 7, Kita-ku, Sapporo, 060-8638 Japan; 2 Department of Physical Therapy, Faculty of Human Science, Hokkaido Bunkyo University, 5-196-1 Kogane-chuo, Eniwa, 061-1449 Japan; 3 Department of Public Health and Hygiene, Graduate School of Medicine, University of the Ryukyus, 207 Uehara, Nishihara, Okinawa, 903-0215 Japan; 4 Research Unit of Advanced Interdisciplinary Care Science, Osaka City University Graduate School of Human Life Science, Sugimoto 3-3-138, Sumiyoshi-ku, Osaka, 558-8585 Japan; 5 Department of Nutritional Epidemiology, National Institute of Biomedical Innovation, Health and Nutrition, 1-23-1, Toyama, Shinjuku-ku, Tokyo, 162-8636 Japan; 6 Department of Preventive Medicine and Public Health, Tokyo Medical University, 6-1-1, Shinjuku-ku, Tokyo, 160-8402 Japan; 7 The Hokkaido Centre for Family Medicine, 1-18, Kita 41, Higashi 15, Higashi-ku, Sapporo, 007-0841 Japan; 8 Suttu Municipal Clinic, 72-2, Utoshima-Cho, Suttu-Cho, Suttu-Gun, Hokkaido, 048-0406 Japan

**Keywords:** Estimated glomerular filtration rate, Physical activity, Sedentary behavior, Accelerometer

## Abstract

**Background:**

Sedentary behavior and decreased physical activity are associated with reduced kidney function, yet most evidence is based on self-reported physical activity. This study investigated the association between accelerometer-based physical activity level and kidney function in a general Japanese population.

**Methods:**

A cross-sectional study was conducted in 440 community-dwelling Japanese participants, aged 35–79 years. Time (min/d) was assessed for the following types of physical activity: sedentary behavior, light physical activity (LPA), and moderate-to-vigorous physical activity (MVPA). Kidney function was assessed using estimated glomerular filtration rate (eGFR). A linear regression model was employed to calculate the β coefficient of eGFR for a 60-min/d increase in sedentary behavior and LPA and a 10-min/d increase in MVPA. A logistic regression model was used to calculate the odds ratio for low eGFR (< 60 versus ≥60 mL/min/1.73m^2^) for a 60-min/d or 10-min/d increase in each physical activity type.

**Results:**

MVPA time and eGFR were positively associated in both men and women, after adjusting for age, body mass index, and other clinical characteristics (Men: *β*, 0.91; *P* = 0.021; Women: *β*, 0.70; *P* = 0.034). In women, sedentary behavior and eGFR were inversely associated after adjusting for the same factors (*β*, − 1.06; *P* = 0.048). The odds ratio (95% confidence interval) for low eGFR associated with a 60-min increase in sedentary behavior was 1.65 (1.07–2.55) after adjusting for the same factors in women.

**Conclusion:**

Longer sedentary behavior and shorter MVPA time were associated with lower kidney function in the Japanese population.

## Introduction

Chronic kidney disease (CKD) is a public health concern worldwide [1, 2]. Previous studies have found that reduced kidney function, even within the range of non-CKD, is a major risk factor for cardiovascular events and deaths independent of classical risk factors, including diabetes and hypertension [3–5]. To reduce this excess burden of CKD, the identification and intervention of modifiable risk factors for the initiation and progression of CKD at an earlier disease stage are important [6].

Decreased physical activity is a modifiable risk factor for cardiovascular disease [7]. Recently, an interest has developed in sedentary behavior that differs from a lack of physical activity, with respect to the risk of cardiovascular disease [8, 9], as well as CKD. Several cross-sectional studies have reported that self-reported sedentary behavior, often independent of reduced time spent engaging in moderate-to-vigorous physical activity (MVPA), was associated with reduced kidney function [10–12]. Although questionnaires assess a variety of physical activity patterns, the use of questionnaires in general is unreliable in assessing the amount of time spent in light physical activity (LPA) and sedentary behavior, leading to the misclassification of inactivity [13, 14]. In this regard, an accelerometer provides a detailed assessment of the quantity and quality of physical activity. However, very few studies have examined the association between accelerometer-based physical activity level and kidney function in the general population of Western countries [14, 15]. This relationship remains unclear in general Asian populations that have different cardiovascular risk profiles compared to the Western populations [16]. Therefore, the present study investigated the association between indices of physical activity measured with an accelerometer and kidney function in a general Japanese population. The accelerometer measured both physical activity intensity and time.

## Methods

### Study design and population

We conducted a cross-sectional study as part of the Dynamics of Lifestyle and Neighborhood Community on Health Study (DOSANCO Health Study), a community-based study in Suttu, Hokkaido prefecture, Japan, from May 2015 to November 2015 [17]. This study invited all residents aged 3 years or above (as criterion for inclusion) in Suttu, except for those living in nursing homes (as criterion for exclusion). Briefly, a total of 2638 residents aged 3–103 years and living at home were considered eligible participants for inclusion, and 2100 participants (977 men and 1123 women) completed a self-administered questionnaire. Of 1379 (650 men and 729 women) participants ages 35–79 for whom we undertook an additional detailed survey, 545 participants (245 men and 300 women) also provided a blood sample. At the same time, these participants were further asked to wear an accelerometer for 14 days.

**Fig. 1:**
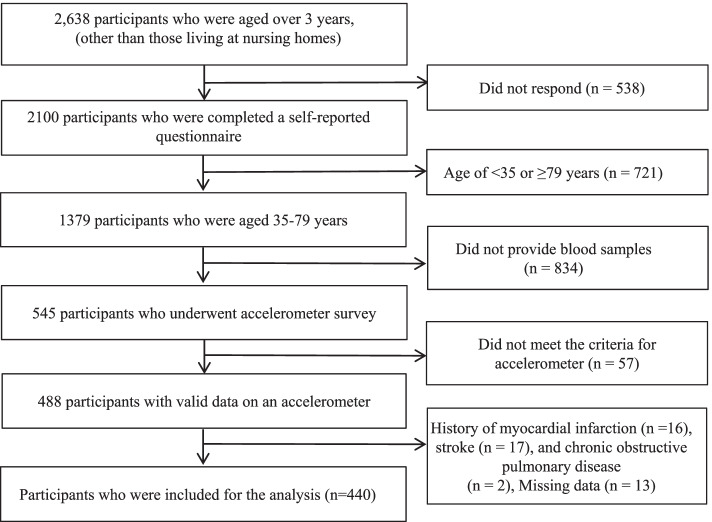
Study flow chart for inclusion of the participants

For the present study, we restricted our analyses to 488 participants who provided valid data on an accelerometer (i.e., 4 days of at least 10 h of consecutive wearing time) [18]. Of the 488 participants, 48 were excluded for the following reasons: history of myocardial infarction (*n* = 16) or stroke (*n* = 17), chronic obstructive pulmonary disease (*n* = 2), and missing data on variables other than accelerometer indices and serum cystatin C level (*n* = 13). Therefore, a total of 440 participants (190 men and 250 women) were included in the final analysis (Fig. 1).

### Assessment of physical activity

An accelerometer (Active Style Pro HJA-750C; Omron Healthcare, Kyoto, Japan) was used to assess physical activity for 14 days. This devise is validated [19, 20], and comparable to other devices commonly used in epidemiological studies [21, 22]. In short, the Active Style Pro has the dimensions of 40 × 52 × 12 mm, weighs 23 g, and records anteroposterior (x-axis), mediolateral (y-axis), and vertical (z-axis) accelerations with a resolution of 3 mG at 32 Hz. Before assessment, the accelerometer was calibrated according to the manufacturer’s recommendations. Each participant wore an accelerometer on the left hip for 14 days while awake, and removed it for water activities and bathing. Non-wear time was defined as 60 mins of continuous zero counts [23], and a standard 60-s data period was used [24, 25]. Valid accelerometer data was obtained if the device was worn for > 10 h/d for at least 4 days [26]. The accelerometer provided data on the intensity of physical activity expressed as metabolic equivalents (METs) using equations for estimation [20, 27]. Time (min/d) was assessed for each of the following three physical activity intensities based on METs: sedentary behavior, ≤1.5 METs; LPA, 1.6–2.9 METs; and MVPA, ≥3.0 METs [20, 28].

### Assessment of kidney function

Blood samples were collected after overnight fast from the antecubital vein of seated participants with minimal tourniquet use. Serum was separated and centrifuged after blood coagulation, and stored at − 80 °C until cystatin C level was measured. Serum cystatin C level was measured on a latex immunoturbidimetric assay using an automated analyzer (JCA-BM8060; JEOL, Tokyo, Japan) in a single laboratory. Estimated glomerular filtration rate (eGFR) was calculated using the Japan Society of Nephrology Chronic Kidney Disease Initiative (JSN-CKDI) equation with serum cystatin C [29]. Serum cystatin C level has an advantage of being less influenced by various factors including age, sex, muscle mass, diet, and physical activity, in contrast to serum creatinine [30].

### Assessment of other factors

Blood pressure was measured twice using an automatic manometer (USM-700G Si; Ueda Avancer Corp., Tokyo, Japan), after the participant rested for 5 min in the seated position. The mean of the first and second readings was used for the analysis. Body height and weight were measured, and body mass index (BMI) was calculated as weight (kg)/height (m^2^). Enzymatic assays were used to measure levels of total cholesterol and high-density lipoprotein (HDL) cholesterol. Non-HDL cholesterol was calculated as total cholesterol minus HDL cholesterol. Hemoglobin A1c (HbA1c) was measured using a latex agglutination immunoassay (Hitachi Chemical Diagnostics Systems Co., Ltd., Tokyo, Japan). Hypertension was defined as either systolic blood pressure ≥ 140 mmHg, diastolic blood pressure ≥ 90 mmHg, or use of antihypertensive medication [31]. Diabetes was defined as either fasting blood glucose level ≥ 126 mg/dL, HbA1c > 6.5%, or self-reported use of diabetes medication [32]. Dyslipidemia was defined as either non-HDL-C ≥ 170 mg/dL or use of medication for dyslipidemia [33]. A self-administered questionnaire collected data, including age, sex, medical history, smoking status, alcohol drinking, dietary intake, and educational background. Smoking status was categorized as “never smoker,” “ex-smoker,” or “current smoker.” Habitual alcohol drinking was categorized as “non-drinker,” “ex-drinker,” or “current drinker.” Dietary intake was assessed using a brief self-administered diet history questionnaire [34]. The data were subsequently converted into total energy intake (kcal/d), fat intake (g/d), and sodium intake (g/d). The education level was categorized as either “high school or less” or “more than high school.”

### Statistical analysis

Data were analyzed separately for men and women due to sex differences in sedentary behavior and physical activity [25, 35]. Initially, we examined the association between time of each physical activity type (i.e., sedentary behavior, LPA, and MVPA; min/d as continuous variables) and eGFR (mL/min/1.73m^2^ as a continuous variable) among all study participants, using a linear regression model. We calculated the *β* coefficient and 95% confidence interval (CI) of eGFR for i) a 60-min/d increase in sedentary behavior, ii) a 60-min/d increase in LPA, and iii) a 10-min/d increase in MVPA [36, 37] using the following models. Model 1 was adjusted for age (years as a continuous variable), accelerometer wear time (min/d as a continuous variable). In contrast, model 2 was adjusted for model 1 variables plus BMI (kg/m^2^ as a continuous variable), smoking status (never, formerly, or currently smoking, using 2 dummy variables with “never smoking” as the reference), alcohol drinking (never, formerly, or currently drinking, using 2 dummy variables with “never drinking” as the reference), energy intake (kcal/d as a continuous variable), fat intake (g/d as a continuous variable), sodium intake (g/d as a continuous variable), educational background (“high school or less” or “more than high school”), hypertension (no or yes), diabetes (no or yes), and dyslipidemia (no or yes). Model 3 further incorporated time of the other physical activity types to assess whether the association of interest was independent of the other physical activity types [36]. Model 3 was adjusted for model 2 plus MVPA time when sedentary behavior and LPA time were examined, whereas sedentary behavior was incorporated as a covariate when MVPA time was examined. Next, we calculated the odds ratio and 95% CI of low eGFR (< 60 versus ≥60 mL/min/1.73m^2^) [38], for a 60-min/d increase in sedentary behavior or LPA, and for a 10-min/d increase in MVPA among all study participants; a logistic regression model that incorporated the same variables as covariates was used. All analyses were conducted using JMP Pro software version 14.0.0 for Macintosh (SAS Institute, Cary, NC, USA). Statistical significance was defined as a two-tailed *P* <  0.05.

## Results

Table 1 shows the characteristics of the 440 study participants together and by sex. In the overall sample, the mean age ± standard deviation was 58.3 ± 12.1 y. The mean length of accelerometer wear was 12 d and wear time was 883.1 ± 90.0 min/d. Mean ± standard deviation of GFR estimated by the JSN-CKDI equation with cystatin C level was 92.6 ± 19.9 mL/min/1.73m^2^, and 5.5% of all participants (5.8% men and 5.2% women) had low eGFR (< 60 mL/min/1.73m^2^). No participants had eGFR < 30 mL/min/1.73m^2^. Women had lower mean BMI, total energy intake, and sodium intake than men. Women were less likely to be smokers and drinkers, had less education, and were more likely to have dyslipidemia than men. In addition, women had a shorter mean time spent engaging in sedentary behavior and a longer mean of LPA and MVPA time than men.

**Table 1:** Demographic and clinical characteristics of the 440 participants from Suttu town, Hokkaido, Japan

	Overall	Men	Women	*p*-value for difference
	(*n* = 440)	(*n* = 190)	(*n* = 250)	
Age (years)	58.3 ± 12.1	57.3 ± 12.1	59.0 ± 12.0	0.13
Body mass index (kg/m^2^)	23.7 ± 3.7	24.4 ± 3.5	23.2 ± 3.8	< 0.001
Smoking status (%)				< 0.001
Never smoker	47.1% (208)	17.4% (33)	70.0% (175)	
Former smoker	32.0% (141)	50.0% (95)	18.4% (46)	
Current smoker	20.7% (91)	32.6% (62)	11.6% (29)	
Alcohol drinking (%)				< 0.001
Never drinker	33.9% (150)	17.9% (34)	46.4% (116)	
Former drinker	8.4% (37)	10.0% (19)	7.2% (18)	
Current drinker	57.5% (253)	71.1% (137)	46.4% (116)	
Total energy intake (kcal/d)	1760.7 ± 539.7	1953.0 ± 546.2	1614.7 ± 487.3	< 0.001
Sodium intake (g/d)	10.9 ± 3.6	11.7 ± 3.8	10.3 ± 3.4	< 0.001
Fat intake (g/d)	49.8 ± 19.1	50.9 ± 18.8	49.0 ± 19.4	0.31
Educational background				0.016
< High school	57.0% (251)	50.5% (96)	62.0% (155)	
≥ High school	43.0% (189)	49.5% (94)	38.0% (95)	
Hypertension	57.7% (254)	57.4% (109)	58.0% (145)	0.89
Diabetes	14.8% (65)	15.8% (30)	14.0% (35)	0.60
Dyslipidemia	34.5% (152)	27.9% (53)	39.6% (99)	0.011
eGFR cystatin	92.6 ± 19.9	92.4 ± 19.3	92.8 ± 20.4	0.82
eGFR< 60 mL/min/1.73m^2^ (%)	5.5% (24)	5.8% (11)	5.2% (13)	0.79
Sedentary behavior (min/d)	463.2 ± 117.5	494.6 ± 119.9	439.4 ± 110.0	< 0.001
Light physical activity (min/d)	369.1 ± 95.6	325.0 ± 83.5	402.6 ± 90.6	< 0.001
Moderate-to-vigorous physical activity (min/d)	50.5 ± 31.1	47.2 ± 29.9	53.1 ± 31.8	0.049

Data are presented for the total study group and also grouped according to sex

Variables are presented as mean ± standard deviation, median (interquartile range), or the % (number) of participants in that category. *eGFR* estimated glomerular filtration rate. Student’s t-test, chi-squared test was used to compare participant characteristics between sex

**Fig. 2:**
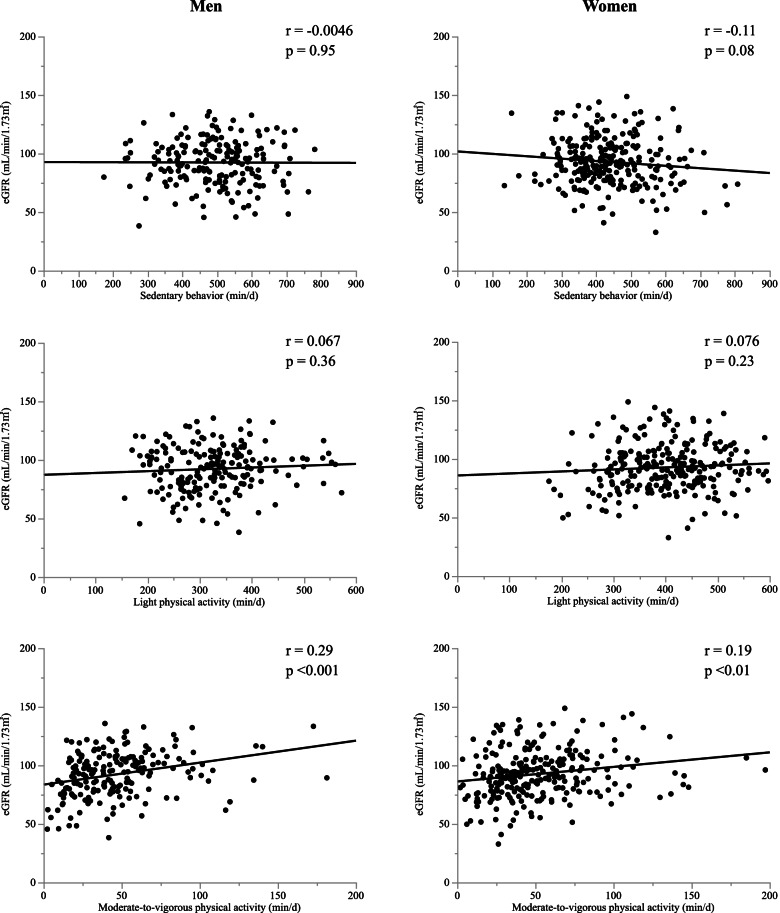
Correlations between sedentary behavior/physical activity and estimated glomerular filtration rate (eGFR) in the study population

Figure 2 shows a sex-specific scatter graph of sedentary behavior, LPA, MVPA, and eGFR. Time in MVPA showed a positive correlation with eGFR in both men and women (men, r = 0.29, *p* <  0.001; women, r = 0.19, *p* <  0.01).

Table 2 shows the *β* coefficients of eGFR for a 60-min/d increase in sedentary behavior and LPA, and for a 10-min/d increase in MVPA in men and women. For both men and women, MVPA time was positively associated with eGFR (Men: *β*, 0.91; 95% CI: 0.14–1.69; Women: *β*, 0.70; 95% CI: 0.055–1.34) after adjusting for possible confounding factors, including age, accelerometer wear time, BMI, smoking status, alcohol drinking, total energy intake, fat intake, sodium intake, educational background, hypertension, diabetes, and dyslipidemia (model 2). This positive association was observed even after further adjusting for sedentary behavior only in men (*β*, 1.02; 95% CI: 0.15–1.90) (model 3). In women, sedentary behavior was inversely associated with eGFR after adjusting for the same potential confounders (*β*, − 1.06; 95% CI; − 2.11– − 0.01) (model 2). This inverse association was attenuated after further adjusting for MVPA time (*β*, − 0.69; 95% CI: − 1.86–0.47) (model 3).

**Table 2:** Linear regression coefficient (95% CI) of eGFR for a 60-min/d increase for sedentary behavior, light physical activity, and for a 10-min/d increase for moderate-to-vigorous physical activity in the 440 participants

	Crude		Adjusted, model 1		Adjusted, model 2		Adjusted, model 3	
	*β* (95% CI)	*p*-value	*β* (95% CI)	*p*-value	*β* (95% CI)	p-value	*β* (95% CI)	*p*-value
Men								
Sedentary behavior (min/d)	−0.04 (−1.44, 1.35)	0.95	−0.37 (−1.45, 0.71)	0.50	−0.34 (− 1.46, 0.77)	0.54	0.33 (− 0.91, 1.57)	0.60
Light physical activity (min/d)	0.93 (−1.06, 2.94)	0.36	0.94 (−0.85, 2.72)	0.30	0.90 (−0.92, 2.72)	0.33	0.36 (−1.51, 2.23)	0.71
Moderate-to-vigorous physical activity (min/d)	1.88 (0.99, 2.77)	< 0.001	0.95 (0.20, 1.70)	0.013	0.91 (0.14, 1.69)	0.021	1.02 (0.15, 1.90)	0.023
Women								
Sedentary behavior (min/d)	−1.23 (−2.61, 0.15)	0.082	−1.22 (−2.25, −0.20)	0.019	− 1.06 (−2.11, − 0.01)	0.048	−0.69 (− 1.86, 0.47)	0.24
Light physical activity (min/d)	1.03 (−0.65, 2.71)	0.23	1.70 (−0.012, 3.39)	0.050	1.03 (−0.71, 2.77)	0.25	0.66 (−1.11, 2.42)	0.47
Moderate-to-vigorous physical activity (min/d)	1.24 (0.46, 2.03)	< 0.001	0.54 (−0.11, 1.19)	0.10	0.70 (0.055, 1.34)	0.034	0.51 (−0.20, 1.23)	0.16

Linear regression model was used to calculate coefficient: Model 1 was adjusted for age and accelerometer wear time. Model 2 was adjusted for the same covariates used in model 1 plus body mass index, smoking status, alcohol drinking, total energy intake, sodium intake, fat intake, educational background, hypertension, diabetes, and dyslipidemia. Model 3 was adjusted for the same covariates used in model 2 plus moderate-to-vigorous physical activity (when sedentary behaviour and light activity were examined) or sedentary (when moderate-to-vigorous physical activity was examined)

Abbreviations: *eGFR* estimated glomerular filtration rate; *CI* confidence interval

Table 3 shows the odds ratios of low eGFR (< 60 mL/min/1.73m^2^) for a 60-min/d increase in sedentary behavior and LPA and for a 10-min/d increase in MVPA among men and women. In women, the odds ratio of low eGFR (< 60 mL/min/1.73m^2^) associated with a 60-min/d increase in sedentary behavior was 1.65 (95% CI: 1.07–2.55), after adjusting for possible confounding factors, including age, accelerometer wear time, BMI, smoking status, alcohol drinking, total energy intake, fat intake, sodium intake, educational background, hypertension, diabetes, and dyslipidemia (model 2). The odds ratio was slightly attenuated at 1.51 (95% CI: 0.94–2.41), even after further adjusting for MVPA time (model 3).

**Table 3:** Odds ratio of low eGFR (< 60 versus ≥60 mL/min/1.73m^2^) for a 60-min/d increase for sedentary behavior, light physical activity, and for a 10-min/d increase for moderate-to-vigorous physical activity in the 440 participants

	Crude		Adjusted, model 1		Adjusted, model 2		Adjusted, model 3	
	OR (95% CI)	*p*-value	OR (95% CI)	*p*-value	OR (95% CI)	*p*-value	OR (95% CI)	*p*-value
Men								
Sedentary behavior (min/d)	1.11 (0.82, 1.50)	0.51	1.36 (0.85, 2.17)	0.21	1.58 (0.88, 2.82)	0.10	1.56 (0.78, 3.13)	0.19
Light physical activity (min/d)	0.81 (0.50, 1.30)	0.37	0.88 (0.46, 1.70)	0.71	0.89 (0.41, 1.93)	0.77	1.07 (0.47, 2.44)	0.88
Moderate-to-vigorous physical activity (min/d)	0.61(0.40, 0.90)	0.023	0.84 (0.58, 1.20)	0.30	0.75 (0.48, 1.16)	0.15	0.88 (0.56, 1.37)	0.55
Women								
Sedentary behavior (min/d)	1.45 (1.09, 1.92)	0.011	1.41 (1.05, 1.94)	0.021	1.65 (1.07, 2.55)	0.013	1.51 (0.94, 2.41)	0.072
Light physical activity (min/d)	0.72 (0.49, 1.07)	0.10	0.60 (0.35, 0.97)	0.037	0.60 (0.31, 1.15)	0.11	0.67 (0.33, 1.36)	0.26
Moderate-to-vigorous physical activity (min/d)	0.72 (0.53, 0.93)	0.0085	0.76 (0.54, 1.00)	0.052	0.69 (0.46, 1.04)	0.036	0.77 (0.49, 1.21)	0.22

Logistic regression model was used to calculate odds ratio: Model 1 was adjusted for age and accelerometer wear time. Model 2 was adjusted for the same covariates used in model 1 plus body mass index, smoking status, alcohol drinking, total energy intake, sodium intake, fat intake, educational background, hypertension, diabetes, and dyslipidemia. Model 3 was adjusted for the same covariates used in model 2 plus moderate-to-vigorous physical activity (when sedentary behavior and light activity were examined) or sedentary (when moderate-to-vigorous physical activity was examined). Abbreviations: *OR* odds ratio; *CI* confidence interval

## Discussion

The present cross-sectional study measured the time spent engaging in sedentary behavior, LPA, and MVPA with an accelerometer among adult residents of a rural community in Japan. Our study showed that time spent engaging in MVPA was positively associated with kidney function (quantified by eGFR with cystatin C) in both men and women, while sedentary behavior time was inversely associated with kidney function in women only. These associations were observed even after accounting for relevant clinical characteristics, including BMI, hypertension, diabetes, and glucose metabolism. Therefore, our study highlighted that the type of physical activity had different associations with reduced kidney function, within clinically normal ranges, for men and women.

On average, people engage in sedentary behaviors for approximately 55% of their awake hours [15]. Many epidemiological studies suggest that sedentary behavior increases the risks of cardiovascular disease and all-cause mortality, independent of obesity [8]. However, limited epidemiological evidence is available on the link between sedentary behavior and reduced kidney function, which is a worldwide public health concern [1, 2]. A previous cross-sectional study involving community-dwelling Japanese older adults showed that individuals who engage in sedentary behavior for longer periods of time (as self-reported) had a higher prevalence of low eGFR of < 60 mL/min/1.73m^2^ [12]. In a United States cohort study, middle-aged participants who participated in MVPA for > 300 min/week (as self-reported) had an 11% reduced risk of incident reduced eGFR of < 60 mL/min/1.73m^2^ over a 24-y follow-up period, compared with those without the corresponding MVPA time [39]. To date, very few studies have examined the association between a reliable measure of sedentary behavior and physical activity and kidney function. The National Health and Nutrition Examination Survey in the U.S. reported that sedentary behavior measured with an accelerometer was inversely associated with eGFR among non-diabetic women [10]. Although men and women were combined in the analyses of these previous studies, the results are partially in accordance with our results for women, but not for men. Meanwhile, a recent cross-sectional study among older adults in the United Kingdom revealed that every 10-min increase in MVPA time had an odds ratio of 0.87 (95% CI: 0.78–0.96) for moderately reduced eGFR of < 45 mL/min/1.73m^2^ [36]. The results of this previous study are partially consistent with our results.

Evidence suggests that physical activity has different beneficial effects on cardiovascular disease in men and women [40, 41]. Similar sex differences may be applicable to renal dysfunction by the following biological differences between men and women. A recent report showed a more positive association between time spent participating in sedentary behavior and pro-inflammatory biomarker levels (such as IL-6 and fibrinogen) in women compared to men [42]. Thus, this report may be similar to our results that sedentary behavior is likely to be associated with reduced kidney function in women only.

Patients with CKD and end-stage renal disease are less physically active and have more sedentary behaviors than healthy individuals [43, 44]. A recent nationwide cross-sectional study reported that, even those with slightly reduced eGFR were more likely to have sedentary behavior than those with normal kidney function (adjusted odds ratio 1.7; 95% CI: 1.2–2.3) [15]. In this study of participants with general population, lower eGFR was associated with decreased MVPA time and increased sedentary behavior. The possible reason for the association between reduced eGFR and less MVPA or increased sitting time is unclear. The Third National Health and Nutrition Examination Survey reported that the incidence of frailty was higher among those with even participants with stage 1 and 2 CKD compared to those without CKD [45]. In addition, a recent cross-sectional study with general population showed that eGFR and skeletal muscle mass per body weight were significantly correlated [46]. It is possible that the physiological changes in the muscles associated with renal dysfunction may decrease physical activity. Meanwhile, according to the 2012 Kidney Disease Improving Global Outcomes (KDIGO) clinical practice guidelines, exercise is recommended for CKD patients [47]. However, there is limited evidence for the benefits of increased physical activity and decreased sedentary behavior for renal function among patients with early-stage CKD, the best candidates who may benefit from healthy behavioral modifications. A recent cohort study in Korea involving 83,470 subjects with early-stage CKD showed that individuals engaged in sufficient physical activity (as self-reported) had significantly reduced risk of death (adjusted hazard ratio 0.73; 95% CI: 0.64–0.83; *p* <  0.001) [48]. The results of our study, which objectively assessed physical activity, are consistent with those of previous studies on patients with early-stage CKD. Although the exact mechanism remains uncertain, longer MVPA time may improve endothelial function of vessels and prevent atherosclerotic change, which may consequently preserve kidney function [49]. Further research is needed to clarify the benefits of increased physical activity for renal function among healthy individuals with normal renal function.

Although our analysis showed that LPA time was not related to renal function, a previous epidemiological study had contrasting results. The study examined the relationship between subjectively measured physical activity and the odds of low eGFR (< 60 mL/min/1.73m^2^), and it suggested that increased LPA time was associated with lower odds of low eGFR [37]. One possible reason for the varied findings between our study and the previous study is the difference in the MVPA time. In the previous studies, the protective effects of increased LPA on mortality were limited to people with low MVPA times [40, 50] . Our study participants had a mean MVPA time of 47.0 min/d for men and 53.1 min/d for women. Thus, we may have observed a null association between LPA time and kidney function for both men and women. It may not be surprising that our results regarding LPA contradict those of a previous study, which showed a positive association between LPA time and kidney function among older adults; the participants had a lower mean MVPA time compared to our study participants [36].

The strength of our study is that we objectively measured sedentary behavior and physical activity using an accelerometer, which allowed us to accurately estimate the time spent in various intensities of physical activity. In addition, we used a novel renal marker (cystatin C) in contrast to creatinine [30, 51] to assess kidney function, and had extensive data on clinical characteristics that were considered potential confounders. However, our study has several limitations. First, due to the cross-sectional design, we were unable to determine the causal nature of the associations observed. Second, due to the low response rate in our study, the results may be biased due to the voluntary participation of individuals who had a high level of health consciousness and physical activity [52]. Third, we did not have data on the use of anti-platelet drugs. However, some participants on anti-platelet therapy may have been excluded to some extent because of the exclusion of those with a history of myocardial infarction or stroke. Fourth, the outcome variable, serum cystatin C levels, was measured once only, and we could not assess urinary albumin excretion. In addition, our study is limited by the small number of participants with low eGFR (< 60 mL/min/1.73m^2^). Finally, our study participants comprised residents of a rural community, where some of residents are engaged in fishing and farming. Since some studies suggest that physical activity differs between rural and urban residents [53, 54], caution is necessary when extrapolating our findings to people in urban areas, as well as general populations.

## Conclusions

Our data demonstrated an inverse association between sedentary behavior and kidney function in women, and a positive association between MVPA time and kidney function in both men and women. Future large-scale longitudinal studies are needed to determine the causal relationship between specific physical activity times and kidney dysfunction, which may help provide a clinical recommendation for various physical activity intensities.

## Data Availability

All data generated or analyzed during this study are included in this article.

## Abbreviations

BMI: Body mass index
CKD: Chronic kidney disease
CI: Confidence interval
DOSANCO: Dynamics of Lifestyle and Neighborhood Community
eGFR: Estimated glomerular filtration rate
HbA1c: Hemoglobin A1c
HDL: High-density lipoprotein
JSN-CKDI: Japan Society of Nephrology Chronic Kidney Disease Initiative
LPA: Light physical activity
METs: Metabolic equivalents
MVPA: Moderate-to-vigorous physical activity
